# Malignant Peritoneal Mesothelioma: A Challenging Case for Palliative Care

**DOI:** 10.7759/cureus.27580

**Published:** 2022-08-01

**Authors:** Carolina Vidal, Inês Romero, Isabel Neto

**Affiliations:** 1 Unidade de Cuidados Paliativos, Hospital do Divino Espírito Santo de Ponta Delgada, Ponta Delgada, PRT; 2 Unidade de Cuidados Continuados e Paliativos, Hospital da Luz Lisboa, Lisboa, PRT

**Keywords:** supportive and palliative care, malignant peritoneal mesothelioma, hyperactive delirium, abdominal pain, malignant obstruction

## Abstract

Malignant peritoneal mesothelioma (MPM) is a rare and very aggressive malignancy of serosal membranes, which typically presents with abdominal pain, distension, and ascites. Due to its rarity and nonspecific symptoms, it is usually diagnosed late, when the disease burden is extensive and the therapy is inevitably palliative. It represents a complex challenge for clinicians because the treatment options are very poor and the illness has a great impact on patients’ life. We present a complex case of a young patient with MPM who was admitted to our palliative care unit.

## Introduction

The literature on malignant peritoneal mesothelioma (MPM) is scarce, particularly regarding the palliative care (PC) approach. We present a case of a young man with extensive MPM in a very complex scenario.

## Case presentation

In June 2019, a 28-year-old male, newly married and Brazilian immigrant in Portugal presented with diarrhea, fever, and ascites. He was diagnosed with an epithelioid peritoneal mesothelioma stage III (T4N1M0), and treated with neoadjuvant chemotherapy (cisplatin plus pemetrexed) until May 2020 and then submitted to extensive cytoreductive surgery (CRS) combined with hyperthermic intraperitoneal chemotherapy (HIPEC).

**Figure 1:**
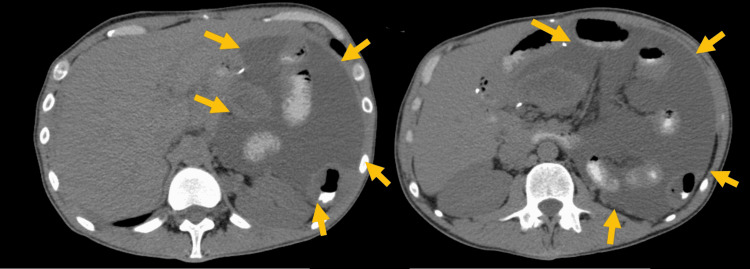
Abdominal computerized tomography showing infiltrative mass with small bowel and colon involvement (arrows).

He was readmitted to the hospital in December 2020, due to abdominal distension, nausea, vomiting, diarrhea, and significant weight loss. Abdominal computerized tomography (CT) showed diffuse wall thickening of almost all colon and some small bowel segments, voluminous infiltrative mass (11 cm) with small bowel and colon involvement (Figure 1). *Clostridioides difficile *infection was diagnosed and treated with vancomycin and then fidaxomicin for recurrence. Although* Clostridioides difficile *toxin was already negative and other infectious agents were excluded, the patient maintained diarrhea, probably linked to disease progression. A naso-jejunal tube was inserted for enteric feeding, paired with parenteral nutrition, in order to improve his nutritional status to try a new chemotherapy treatment.

The patient was discharged, maintaining enteric feeding, but had to be readmitted early in January due to abdominal pain, distension, and diarrhea. CT scan showed significant disease progression determining gastric distension and duodenal compression. Parenteral nutrition was resumed since no enteric feeding was tolerated and the patient was submitted to another chemotherapy treatment (cisplatin plus pemetrexed). The patient experienced persistent symptoms, namely abdominal pain and distension, managed with continuous nasogastric drainage in the context of upper malignant bowel obstruction (MBO). Despite loperamide treatment, watery diarrhea (eight dejections per day) was also present.

The patient requested hospital transfer and was admitted to our PC unit on January 28th, 2021. On admission, he was able to walk with assistance but was unable to perform effortful tasks, scoring 50-60% on the Palliative Performance Scale [1]. His young wife was allowed to stay with him, despite the COVID-19 restrictions. On admission, he was cachectic, had persistent gastric drainage via nasogastric tube (NGT) placed, and his abdominal exam showed non-tense ascites and dispersed abdominal masses.

Symptom management was then optimized. Diarrhea and abdominal pain were controlled with low-dose morphine and gastric drainage was significantly reduced with hyoscine butylbromide (HBB). A NGT was maintained, and the patient ingested some food and drinks - water, juices, ice cream - aiming for comfort.

Despite symptom improvement, progressive decline ensued in the following days, and for this reason, he was deemed to not be a chemotherapy candidate. Daily conversations with the patient and his wife were required in order to adjust them to this transition of care.

As the patient began to realize the severity of his disease and short-term prognosis, he became restless, mainly because of his family’s absence, due to COVID-19 restrictions. His mother managed to fly from Brazil and visit due to the patient's health condition. His young wife received psychological counseling. The patient became severely anguished, with hyperactive delirium, and palliative sedation (levomepromazine and midazolam) was started. He died peacefully late on the 9th of February, with his wife at his bedside.

## Discussion

Due to the nonspecific nature of the initial symptoms, many patients with MPM present with advanced disease at diagnosis [2]. The main initial symptoms are abdominal pain, distension, and ascites [3,4]. As the disease progresses, patients can develop nausea and vomiting, constipation, diarrhea, anorexia, weight loss, and dyspnea [2,5]. MBO is usually a manifestation of advanced disease [2].

Oncologic treatment

Due to the rarity of MPM, most of the information about the treatment comes from retrospective studies [6]. In a multi-institutional series of 401 patients, those submitted to CRS and HIPEC had the most favorable outcomes [4]. For patients with diffuse MPM, without extraperitoneal disease, with good performance status, and predicted to achieve complete CRS, most of the experts recommend CRS and HIPEC [4-7]. Among centers with expertise in CRS and HIPEC, reported median survival approaches five years [6].

Systemic chemotherapy is an alternative approach for inoperable patients [5,7]. Perioperative systemic chemotherapy is also recommended in those with high-risk histology or extensive disease, which was our patient’s case [5]. Pemetrexed monotherapy has an estimated median overall survival of 8.7 versus 13.1 months for pemetrexed and cisplatin combination therapy. Therefore, the current standard is doublet therapy [7]. Particularly in the palliative setting, pemetrexed plus carboplatin or pemetrexed plus gemcitabine are safe alternatives [6,7].

Importantly, in young patients, we usually observe high aggressiveness in oncological treatment. This is frequently linked with the oncologists’ perception of the unfairness of dying young, over-identification, and emotional entanglement [8].

Supportive treatment

Abdominal Pain

Opiates provide adequate pain relief for somatic and visceral pain. Adjuvants such as corticosteroids and antispasmodics are also useful. Given the constipation potential, in this case, we rotate from transdermal fentanyl to subcutaneous (SC) morphine. We used dexamethasone as a co-analgesic because of its important role in MBO and liver capsule distension pain.

Malignant Bowel Obstruction

In patients previously treated with chemotherapy, MBO represents a poor prognosis, usually less than 60 days of expected survival [9]. In upper MBO, the nausea is intense and presents early, the vomiting is copious, and pain and distension are usually absent [10]. In lower MBO, distension and pain predominate, and vomiting usually occurs later and can be fecaloid [10].

Surgery should be avoided in elderly age, cachexia, multilevel obstruction, refractory ascites, or absence of specific oncologic treatments [10]. Some patients not suitable for surgery can benefit from endoscopic stenting of gastric outlet, colon, or rectum obstructions [10,11]. Multilevel disease, peritoneal carcinomatosis, and low-performance status contraindicate endoscopic procedures [10].

Given the multilevel obstruction and cachexia, surgery or endoscopic procedures were not advisable in this case. Indeed, medical management represents the mainstay intervention for symptomatic MBO [11]. We introduced dexamethasone 16 mg/day (SC) in order to reduce peri-tumoral and intestinal edema [10,11]. Metoclopramide given SC (40 to 120 mg in SC continuous infusion - CSCI) can also help to revert MBO [10], but in irreversible occlusions, metoclopramide causes colicky pain and should be avoided [10,12]. In this case, diarrhea and high gastric drainages contraindicated the use of metoclopramide. 

In irreversible MBO, HBB can reduce intestinal peristalsis and secretion due to its antimuscarinic effect [10], providing pain relief and reducing diarrhea. HBB can be used by intravenous (IV) or SC route, in bolus or continuous infusion, and can be titrated up to 120 mg/day by CSCI [10,12]. Herein, HBB 60 mg/day/SC was introduced in order to control pain, diarrhea, and gastric drainages.

Somatostatin analogs such as octreotide inhibit gastric, pancreatic, and intestinal secretions, and can help to relieve pain, nausea, and vomiting in MBO [10,11]. Octreotide may be effective in MBO patients in whom HBB has failed. The usual starting dose is 0.1 mg twice daily SC, but rapid titration may be needed up to 0.9 mg/day [10]. Patients who respond can receive a depot injection of long-acting octreotide monthly for maintenance therapy [11].

Haloperidol is a potent suppressor of the chemoreceptor trigger zone [10] and is often used in MBO; it can be administered SC as a bolus or as a CSCI. Levomepromazine, due to its potent sedative effect, is reserved for refractory symptoms [13].

Importantly, in MBO, NGT should be only a temporary measure. Exceptionally in this case due to the high volumes of gastric drainage refractory to drug therapy, NGT was maintained. During the treatment with HBB, it was possible to frankly reduce the gastro-intestinal drainages, but nausea rapidly ensued when the tube was clamped. When the removal of the NGT is not possible, venting gastrostomy is a reasonable longer-term alternative [11]. In our patient, with a very advanced disease, an endoscopic procedure was deemed too invasive.

Nutrition

During the patient’s stay, we resumed parenteral nutrition. Although refractory cachexia is linked to advanced disease, the patient had high expectations of resuming artificial nutrition and chemotherapy. To ensure patient’s adherence and start the process of adjusting expectations we underwent artificial nutrition in the first days. We also encouraged our patient to drink liquids of his preference, which had an important impact on his overall well-being. Oral hydration with fluids helped with dry mouth complaints and thirst. Topical solution with bicarbonate, lidocaine, and nystatin also helped with mucositis. The patient became progressively bedbound, with diminished interaction with the external environment. Accordingly, we reduced and then stopped the parenteral nutrition, after adequately framing the decision with his family.

Terminal Delirium

More than 90% of patients with cancer experience terminal delirium. Despite the treatment of reversible causes and non-pharmacological measures, many patients will need pharmacological interventions [14].

There remains no definite evidence that antipsychotics reduce delirium duration or severity [15]. Despite this, haloperidol (1-30 mg/day/SC) still is the drug of choice for hyperactive delirium [13,16]. Levomepromazine (6.25 mg-300 mg/day/SC) is another option for hyperactive delirium [13,17]. It shows a more sedative effect and longer half-life than haloperidol.

Benzodiazepines should be used when severe agitation is refractory to antipsychotics. Midazolam has a rapid onset of action, allowing for rapid control of agitation. Doses can range 1-20 mg/h IV or SC route [17].

Our patient developed hyperactive delirium, initially managed with haloperidol, but when agitation increased, continuous palliative sedation was initiated with levomepromazine and midazolam. 

Relevance of PC for MPM patients

Literature on MPM and PC is very scarce. Most information on this subject comes from studies on pleural mesothelioma [18,19]. Given the high symptom burden in MPM, PC should be integrated earlier in the approach for these patients. Unfortunately, in most cases, PC is left to the last weeks or days of life [19,20]. Indeed, a study that evaluated PC referral of patients submitted to CRS and HIPEC showed that the median time to PC referral was 310 days from the original consultation with a surgical oncologist [20].

In contrast, another study showed that providing early PC to all newly diagnosed patients with pleural mesothelioma was not associated with beneficial changes in quality of life when compared to PC review based on symptom burden [18]. Probably the greatest benefit in quality of life should be noticed when PC referral is based on symptom burden, rather than an automatic referral.

## Conclusions

The clinical presentation and treatment options described in this case do not differ from pre-existing literature on MPM. This case stands out due to the various complex criteria (young age, immigrant, MBO, and decisions regarding artificial nutrition) that highlight the need for an integrative PC approach.

Literature on MPM and PC is very scarce. Most information comes from studies on pleural mesothelioma. Unfortunately, our conclusions are in agreement with other studies that have identified a late referral of patients with pleural mesothelioma and patients submitted to CRS and HIPEC for PC. 

PC promotes better symptom control, enhances patient and family satisfaction, and reduces inappropriate therapeutic interventions toward the end of life. The findings of our case support the need for an earlier PC approach for MPM patients with a high symptom burden.
